# Harnessing Deep-Hole Drilling to Fabricate Air-Structured Polymer Optical Fibres

**DOI:** 10.3390/polym11111739

**Published:** 2019-10-24

**Authors:** Eneko Arrospide, Iñaki Bikandi, Igor Larrañaga, Xabier Cearsolo, Joseba Zubia, Gaizka Durana

**Affiliations:** 1 Department of Applied Mathematics, University of the Basque Country, 48013 Bilbao, Spain; 2 Department of Communications Engineering, University of the Basque Country, 48013 Bilbao, Spain; inaki.bikandi@ehu.eus (I.B.); joseba.zubia@ehu.eus (J.Z.); gaizka.durana@ehu.eus (G.D.); 3 IMH Advanced Centre in Manufacturing, 20870 Elgoibar, Spain; ilarrana@imh.eus (I.L.); cearsolo@imh.eus (X.C.)

**Keywords:** fabrication of polymer preform, microstructured polymer optical fibres, deep hole drilling, poly(methyl methacrylate), thermoplastics, hole quality parameters

## Abstract

The performance of a precisely controlled drilling technique is critical in the fabrication process of microstructured polymer optical fibres. For the creation of a holey preform, adequate drilling bits with large length-to-diameter ratios provide the ability of machining preforms with complex structures and large lengths in a relatively short time. In this work, we analysed different drilling bits and techniques that can be employed for the creation of such preforms, and key parameters characterising the quality of the drilled holes, such as surface rugosity, diameter deviation, coaxiality and cylindricity were measured. For this purpose, based on theoretical simulations, four rings of air holes arranged in a hexagonal pattern were drilled in the preforms with different drill bits, and the experimental results for the above mentioned parameters have been presented. Additionally, optical power distribution of the fabricated microstructured polymer optical fibres was theoretically calculated and experimentally measured.

## 1. Introduction

Microstructured polymer optical fibres (mPOF) can be fabricated with almost any arbitrary pattern of microholes along the longitudinal axis of the fibre, offering the potential for fabricating fibres with a broad range of geometries and applications [1,2]. Although the concept of guiding light through the internal microstructure of such fibres was first explored in silica in photonic crystal fibres (PCFs) [3,4,5], using transparent and drawable polymer materials instead of silica in the fabrication of this type of fibre provides a wider range of mechanical and optical properties, with excellent performance in sensing applications [6,7]. Traditionally, mPOFs have been fabricated using thermoplastics such as poly(methyl methacrylate) (PMMA), polycarbonate (PC) or the fluorinated polymer CYTOP. Copolymers such as TOPAS cyclic olefin copolymer have also attracted the attention of the international research community [8,9,10,11,12].

Compared to silica based PCFs, where the holey structure is mostly limited to hexagonal or square close-packed structures due to the commonly used capillary stacking techniques [13,14], in the case of mPOFs, polymer preforms can be created using several techniques, such as extrusion, moulding, drilling or 3D printing [6,15,16,17], and therefore, structures with holes of arbitrary shapes and sizes can be achieved. Another advantage of using polymers instead of silica in the fabrication of microstructured fibres is related to their mechanical properties. While the viscosities of drawable polymers and silica are of similar magnitude at their respective drawing temperatures, the surface tension of polymers is an order of magnitude lower than that of silica, thus minimizing the hole distortion and collapse due to surface tension effects [2].

Drilling is the most commonly used method in the fabrication of mPOFs. Using a computer numerical control (CNC) machining centre and deep hole drilling bits [18,19,20,21,22], a large number of hole arrangements and hole sizes can be drilled with high precision over a thick polymer rod using an automatised process. Hole arrangement depends on the final application of the fibre. As an example of great practical interest, ring-like hole arrangements are used for single-mode operation in mPOFs [23]. The relatively low glass transition temperature (*T*_g_) of polymers, in the range of 110 °C to 150 °C, and their low molecular weight together with a low thermal conductivity [24] imply that they can be easily overheated [25,26]. For that reason, drilling parameters such as cutting speed, spindle speed, feed or depth of cut must be precisely selected in order to obtain high quality geometries and a fine surface finish in the inner surface of the holes. Nowadays, the most commonly used drill bits for preform creation are high-speed steel bits, and their inability to properly remove the chips for high depths of cut requires the bit to be lifted from the hole continuously, making the drilling a time-consuming process.

In this paper, we analysed and compared the use of standard high-speed steel bits with alternative through-coolant deep-hole drill bits, with the aim of reducing the time required to create holey structured preforms, as well as providing high quality and high precision structures. For this purpose, the drilling of PMMA rods with different kind of drill bits has been described. The quality of the obtained structures was analysed in detail, measuring the variations in the diameter of the holes, their cylindricity, coaxiality and the roughness of the inner walls. Finally, an mPOF consisting of four rings of holes was manufactured and optically characterised to assess the validity of using deep-hole drilling as an improved alternative to standard high-speed steel bits.

## 2. Materials and Methods

### 2.1. Materials

Two essential properties that any polymer must fulfil for the manufacture of microstructured optical fibres are compatibility with the fibre drawing process [1], and a high transparency in the wavelength region of interest. Among the different available thermoplastic polymers that fulfil these requirements, PMMA has been extensively used for the fabrication of microstructured polymer fibres due to its excellent light transmittance in the visible region and its availability and low cost [27]. Therefore, we chose extruded PMMA rods commercialised by Evonik (Essen, Germany) [28] as the starting bulk polymer for machining a given pattern of holes. This kind of rod, unlike most rods prepared by casting or moulding, presents a low average molecular weight and low degree of cross-linking, allowing the polymer to flow smoothly above its *T*_g_, thus avoiding a rubbery behaviour [29]. However, the polymer’s low thermal conductivity of 0.2 W·m^−1^·K^−1^ [30] and its high thermal expansion coefficient around 9 × 10^−5^ K^−1^ [6] makes accurate machining operations difficult, because these parameters tend to increase the friction, and therefore the heat generation, between the machining tool and the polymer. For that reason, adequate drill bits that inject cooling, facilitate chip ejection, prevent polymer melting and ensure smooth surfaces must be used, together with a wise selection of cutting parameters and processing methods.

### 2.2. Drilling Bits

Drilling the preform involves the creation of a large-scale version of the desired microstructure in the final optical fibre. For that purpose, the preform drilling method used must ensure that the quality of the drilled holes, which is related mainly to surface smoothness and hole shape and straightness, is good enough to minimise any negative effects on the optical properties of the drawn fibre [2]. At this point, it is important to bear in mind that in many applications, complex microstructure geometries are required. Those geometries rely on the creation of a large number of small diameter holes within the cross-section of the optical fibre, sometimes with thin bridges separating the air holes. In addition to that, long preforms are preferred in order to reduce the proportion of wasted polymer that inevitably occurs during the drawing stage; thus, long drilling bits are highly prioritised.

Considering the above-mentioned mechanical constraints as well as the time-consuming process of preform drilling when standard drill bits are used, the convenience of using through-coolant deep-hole drill bits with a large length-to-diameter ratio was thoroughly analysed.

**Figure 1:**
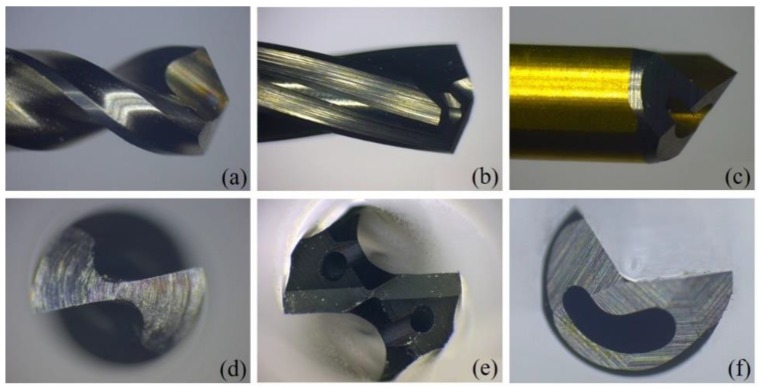
Side (top row) and front (bottom row) views of the three drill bit types employed in this work: (**a**,**d**) a high-speed steel twist drill (HSTD), (**b**,**e**) a through-coolant carbide twist drill (TCCD) and (**c**,**f**) a single-lip deep-hole drill (SLD).

The experimental study was carried out using three types of drilling bit: a high-speed steel twist drill (HSTD), a through-coolant carbide twist drill (TCCD) and a single-lip deep-hole drill (SLD). Front and lateral image views of each type of bit are shown in Figure 1.

Drilling a hole straight through a PMMA rod with a HSTD bit produces long spiral-like chips as they exit along the flute of the drill. These chips scratch the holes, which is detrimental in terms of roughness quality and makes the entrance of the coolant fluid difficult, increasing the heat in the tip of the tool. The generated heat tends to melt the polymer, which then sticks to the drilling tool and provokes its break. For this reason, when HSTD bits are employed, it is common practice to use the pecking method [20,31]. In this technique, the bit is fed to a predetermined depth and then retracts to a level above the preform surface. The cycle consisting of these two steps repeats continuously, but from one cycle to the next the tool is fed to drill a certain additional depth until the through-hole is achieved. The selected depth distance is chosen to produce chips small enough in size to facilitate their continuous extraction and avoid chip stacking within the hole. With this technique, the external coolant is also another important factor to bear in mind. The coolant cleans and cools down the drilling tool, and also refreshes the hole in each extraction to guarantee minimum friction between the bit and the chips. The main drawbacks of this method are that preform fabrication becomes an extremely time-consuming process, and that the smoothness of the inner hole walls is not completely guaranteed.

With the aim of overcoming those limitations and therefore avoiding the use of the pecking method, we investigated the effect of using through-coolant TCCD and SLD bits for the fabrication of the macroscopic polymer preform. In the former case, the coolant is supplied to the cutting edge through the ducts that go through the drill bits following a helical path. The coolant reaches the cutting edge, breaks the chips and evacuates them via the flutes by pushing the chips up along the helical trajectory. In order to ensure the cleanliness of the fluids and avoid blocking the cooling ducts and subsequent breaking of the bits, swarf and other contaminants must be filtered in size, avoiding the recirculation of particles above 8 microns.

The third kind of drilling bit employed, SLD bits, have an asymmetrical design, with a V-shaped external flute and guide pads. The guide pads provide a self-guiding effect and therefore improve the surface smoothness of the inner wall of the holes [32]. In this kind of bit, the cooling lubricant is supplied through the straight coolant channel in the core of the drill, and the removal of the mixture of chips and cooling lubricant takes place along the straight, V-shaped external flute.

In our experiments, four drill bits were employed:

- A standard twist drill of 3 mm diameter and with a length-to-diameter ratio of 40 (HSTD3) [33].
- A through-coolant carbide drill of 3 mm diameter and with a length-to-diameter ratio of 30 (TCCD3) [34].
- A single-lip gundrill of 2 mm diameter and with a length-to-diameter ratio of 50 (SLD2) [35].
- A single-lip gundrill of 3 mm of diameter and with a length-to-diameter ratio of 50 (SLD3) [35].

### 2.3. Drilling Procedure

**Figure 2:**
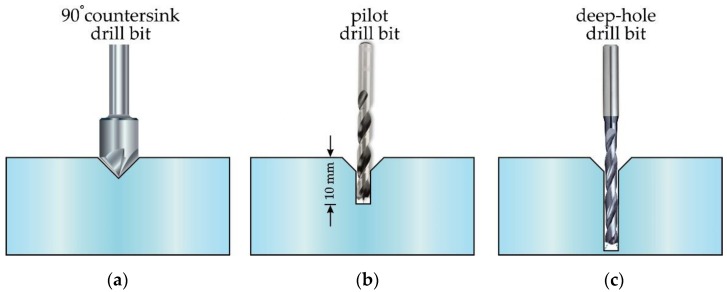
Sequence of steps followed during the drilling process of the preforms. (**a**) Countersink drill bit, (**b**) pilot drill bit and (**c**) final deep-hole drill bit.

Each of the drilling bits described in the previous subsection was used to drill holes in a solid PMMA preform of 60 mm diameter. The drilled structure followed a specific geometry that consisted of a set of 60 air holes arranged in a hexagonal distribution of four rings. The number of rings determines the confinement loss. For the case of single-mode propagation, confinement loss reduces exponentially with the number of rings [36]. By contrast, in the context of Bragg gratings inscribed into mPOFs, only two-three rings are usually used, as the thick cladding region makes the inscription of the grating difficult [37]. A computer numerical control (CNC) multiprocess machining centre Ibarmia ZVH 38-L1600 (Ibarmia, Azkoitia, Spain) was used and the workpieces were fixed in a three-jaw chuck plate. At each hole position, and before drilling the corresponding hole straight through the preform, a set of steps were carried out in order to achieve a repetitive hole drilling process that provided excellent values for the quality parameters described later in the text (see the sequence of steps illustrated in Figure 2). First, a face milling drill bit was used to flatten the top surface of the preform; a 90° countersink drill bit then created a conical hole that acted as a spot drill for the pilot drill bit that came next. The pilot drill bit was a carbide drill with six guide chambers, internal cooling channels and a 140° point angle. The small hole drilled with this pilot drill bit—10 mm in depth—guided the final drill bit in a straight trajectory, minimizing hole deviations due to hole length and material softness. Finally, the deep-hole drilling bit came into operation. In the described drilling procedure, common devices in deep-central-hole drilling like guide bushings were skipped in order to avoid slowing down the process.

**Figure 3:**
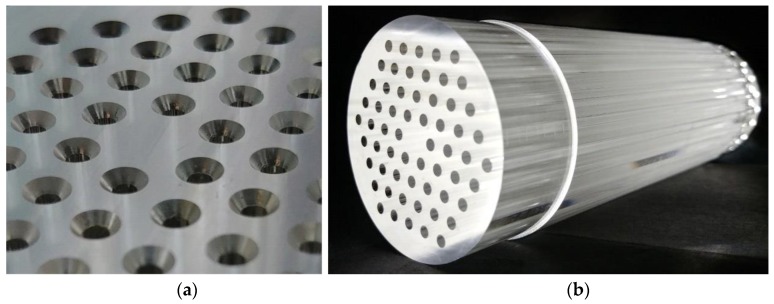
(**a**) Image of the top end of the preform, in which the conical profiles can be observed. (**b**) Bottom end of the preform, where the four rings of 60 through-holes are shown.

As an example of the above explained procedure, Figure 3a shows an image of the result obtained after drilling the conical holes, whereas Figure 3b shows the final result after drilling the sixty through-holes in the PMMA.

In order to avoid undesirable effects like drill overheating, burning the polymer or roughening the inner walls of the polymer, the drilling parameters were tuned according to the mechanical and physical properties of a soft material like PMMA. Additionally, due to the large length-to-diameter ratio of the SLD drill bits, lower speeds were employed with these drill bits. The most significant drilling parameters used are summarised in Table 1:

### 2.4. Quality Parameters

**Figure 4:**
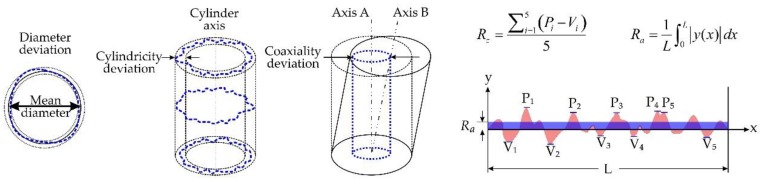
Schematic representation of the parameters defined to evaluate the quality of the holes. From left to right: diameter deviation, cylindricity deviation, coaxiality deviation and roughness. *P*_i_: profile peak; *V*_i_: profile valley (i ∈ [1, 5]).

In order to empirically evaluate the quality of the drilled holes, different parameters were analysed. Specifically, four quality parameters were measured and analysed: the diameter deviation (DD), cylindricity deviation (CYD), coaxiality deviation (COD) and surface roughness (SR). Their definitions are illustrated in Figure 4.

DD refers to the variation in size, formally defined as the difference between the measured hole diameter and the nominal diameter of the bit; thus, a positive error indicates overcutting of the material. CYD represents the difference in radii between two coaxial cylinders constructed to touch and enclose the extracted cylindrical surface with minimum separation. COD is defined as twice the distance between the extracted cylindrical surface axis and the datum axis over the length of the drilled holes. Finally, SR is another important quality characteristic that represents the random and repetitive deviations of the inner surface of the holes. Two values used to quantify the surface roughness are roughness average (*R*a) and average maximum height of the profile (*R*z). The former is the arithmetic average of the absolute values of the profile heights over the evaluation range, whereas the latter is defined as the average value over the absolute values of the five highest profile peaks (*P*_i_) and the five deepest valleys (*V*_i_) within the evaluation length.

### 2.5. Measurement Technique

DD, CYD and COD were measured using a Mitutoyo Coordinate Measuring Machine 7106 (Mitutoyo, Kawasaki, Japan). Each of the 60 holes was analysed in three transverse sections, specifically located at 11, 15 and 20 mm from each of both endfaces (top endface and bottom endface) of the workpiece. For each transverse section, five points evenly spaced along the inner wall of each hole were explored. SR measurements were carried out using the Mitutoyo Surftest SJ-500 Surface Roughness Tester (Mitutoyo, Kawasaki, Japan). When the tester is right over the preform endface, the stylus starts moving along the hole to measure the roughness over a distance of 4 mm. For each of the four quality parameters, in order to make sure that the collected experimental data were related to the drilling bits under study and not to the pilot drill, the first 10 mm from each end surface of the preform was disregarded from the measurement.

## 3. Results and Discussion

### 3.1. Experimental Results

In this section, we present and discuss the results of the quality parameters (DD, CYD, COD and SR) measured for each of the four drill bits used. Having fixed the drill bit and the preform endface, each quality parameter has been plotted using a bar chart and the associated error bar. The bar represents the average value over a data set of 60 values, and the vertical error bar shows the standard error of the mean.

**Figure 5:**
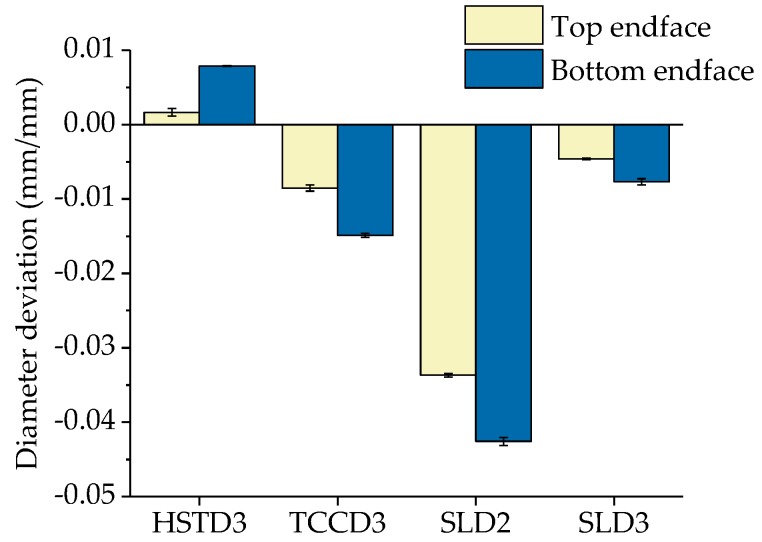
Average value and standard error of the mean of the quality parameter diameter deviation (DD) obtained for each of the drilling bits and endfaces.

The experimental results corresponding to DD are shown in Figure 5. DD values reported in the graphs have been normalised with respect to the diameters of the drill bits. We observed that HSTD3 presented a positive deviation, which means that the measured diameter of the holes was bigger than the diameter of the drilling bit. By contrast, the measured hole diameters for TCCD3, SLD2 and SLD3 were smaller than the nominal diameter of the corresponding bits. This behaviour could be explained on the basis of the temperature reached on the hole surface while drilling. The pecking method used with HSTD3 required an intermittent advance in order to avoid polymer overheating. In contrast, in the case of TCCD and SLD, the drilling of the complete hole was carried out in a single continuous advance, so that the contact time of the drilling bit with the material substantially increased, eventually yielding a higher cutting temperature. The higher heat in the holes while drilling could have provoked an expansion of the polymer and consequently, a reduction in the diameter of the holes. Additionally, drilling the smallest holes using SLD2 presented the greatest expansion and reached a diameter deviation around 4%, while diameter deviation stayed below 1.5% for the bigger SLD3 and TCCD3. This behaviour could be the consequence of poor cooling, and therefore higher thermal expansion occurring in the preform when using the 2 mm drill bits.

It is also worth pointing out that the uncertainty of the measurements was minimal and practically negligible for all through-coolant drill bits (SLD2, SLD3 and TCCD3), an unconditional requirement needed to support a high reproducibility of the drilling process.

**Figure 6:**
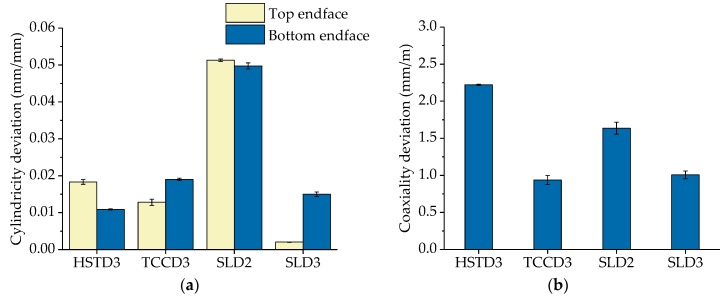
Average value and standard error of the mean of the quality parameters (**a**) cylindricity deviation (CYD) and (**b**) coaxiality deviation (COD) for each of the tested drilling bits.

The experimental results corresponding to the parameters CYD and COD are shown in Figure 6. With respect to CYD, it must be remarked that measured values have been normalised with respect to the diameters of the drill bits. It was observed that the largest deviation corresponded to SLD2, with a deviation value of around 5% reached at both ends of the preform (see Figure 6a). Regarding the rest of the drilling bits, the deviation never exceeded 2%. As in the case of DD, this behaviour could have been the consequence of a higher thermal expansion occurring in the preform when using the smallest cutting tool. On the other hand, regarding COD, Figure 6b shows the experimental values of COD normalised with respect to the length of the corresponding preform. The worst results were obtained for HSTD3, with a normalised COD value above 2.2 mm/m. For the other cases (through-coolant drilling bits), the value was always below 1.7 mm/m. These results can be explained in terms of the lower rigidity of the former tool (HSTD3) compared to the carbide ones (SLD2, SLD3 and TCCD3), which resulted in a higher tendency to deflect during the drilling process. COD values as small as possible of are of primary importance for the subsequent preform drawing process that follows, as they will provide stability to the fabrication process and minimum deviation from the translational symmetry required for optical fibres.

**Figure 7:**
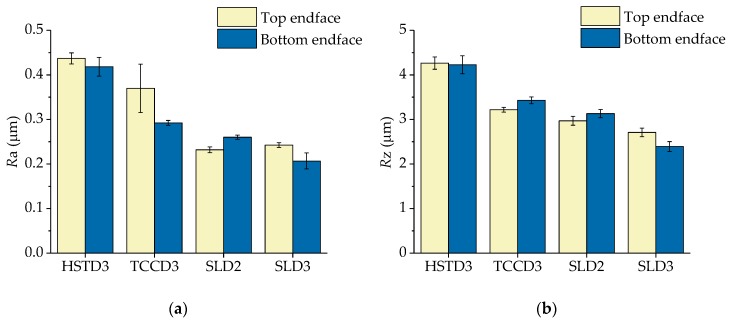
Average value and standard error of the mean of the quality parameter surface roughness (SR) quantified as (**a**) roughness average (*R*a) and (**b**) average maximum height of the profile (*R*z).

Finally, the results corresponding to the SR quality parameters are shown in Figure 7, where both *R*a (Figure 7a) and *Rz* (Figure 7b) are plotted. As expected, the worst results corresponded to the holes drilled with HSTD3. The pecking method implies an intermittent and repetitive advance/retraction of the drill all along the preform, thus resulting in unavoidable marking of the inner surface of the holes as a consequence of the advancing/retraction movement of the cutting tool, and the resulting continuous rubbing. The lack of guiding pads of HSTD3 also promoted a higher SR. When using the other drill bits (drills with inner coolant supply), which included guiding pads and with which the drilling process was executed in a single cycle, SR results were improved. More specifically, *R*a values remained in the range between 0.2 and 0.35 μm, and *R*z values between 2.5 and 3.5 μm. The best results were obtained with the SLD. For this type of drill, the chips flow upwards through a straight channel, in contrast to TCCD3, for which the chips evacuate following a helical trajectory.

Having analysed the influence of different kinds of drill bit on the drilling process, another parameter of great importance that cannot be overlooked is the time required to create the structured preform. Table 2 compares, for the given geometry, the overall fabrication time for each of the drill bits used. The pecking method used with HSTD bits represented, by far, the most time-consuming technique. In contrast, TCCD was the fastest one, being 15 times faster than the slowest one. The drilling time with SLD bits fell in between these cases, but was in close proximity to the TCCD performance. In relation to SLD bits, it must be pointed out that the drilling speeds were chosen to ensure that they were low enough to be on the safe side. However, they could be further increased, yielding substantially faster fabrication times. It is also important to point out the maximum preform length achievable with each of the drill bits, for which SLDs led the ranking. For the case of 3 mm diameter drill bits, the structured preforms were 46% longer than those obtained using HSTD bits combined with the pecking method, and 88% longer than those fabricated with TCCD bits. Therefore, SLDs represent the best trade-off between fabrication time and preform length among the different choices tested herein.

### 3.2. Fabrication and Assessment of Optical Guidance Properties of the mPOF

With the aim of validating the proposed preform drilling technique for the fabrication of mPOFs, we transferred the geometry described previously to a solid PMMA preform using SLD3 bits. As a first step of the overall fabrication process, we carried out a theoretical analysis of light propagation in the microstructure of interest. Subsequently, a scaled-up version of the desired holey microstructure was drilled in a solid preform and then drawn to fibre following a two-step process described elsewhere [38]. Finally, the near-field pattern of the fabricated mPOF was recorded and compared with that obtained in the first step (theoretical analysis).

**Figure 8:**
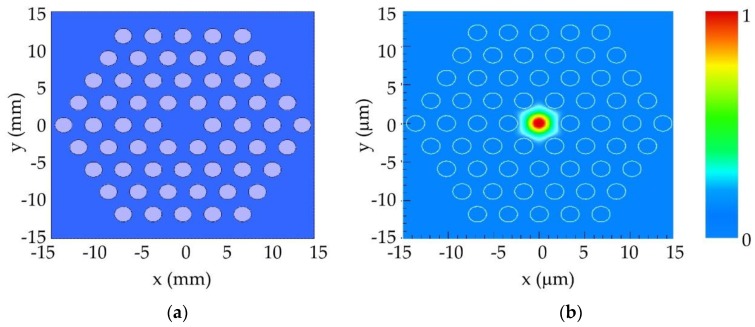
(**a**) Geometry of the microstructure considered for the computer simulations. (**b**) 2D plot of the simulation results for the near-field pattern, which shows the light confinement within the core associated to the unique guided mode.

**Figure 9:**
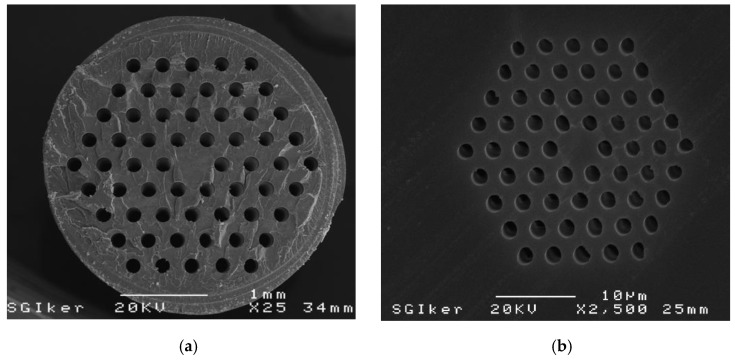
Scanning electron microscope (SEM) images of (**a**) the intermediate cane and (**b**) the core/cladding region of the fabricated microstructured polymer optical fibre (mPOF).

The theoretical modelling of the selected microstructure relied on computer simulations carried out in a fully vectorial mode solver included in the FIMMWAVE suite from Photon Design^®^. It is worth noting that the simulation results represent only an approximation to what really happens in terms of light propagation inside real mPOFs, because they assume a perfect geometry of the microstructure that does not actually happen. Small hole deviations occur during the drilling stage of the solid preform, and the holey structure is additionally deformed during the two-stage drawing process [38]. The microstructure shown in Figure 8a corresponds to the selected fibre geometry, consisting of a central core of 5 μm in diameter and surrounding air holes of 2 μm in diameter. The corresponding simulated near-field pattern is shown in Figure 8b. It was observed that light is almost completely guided in the core region, and spatial power distribution decreased sharply in the core/cladding interface. Setting this theoretical case as a useful reference point for future comparison, an up-scaled version of this microstructure was drilled in a 60 mm diameter PMMA solid preform, following the drilling procedure described previously for SLD bits. The scanning electron microscope (SEM) photograph shown in Figure 9a shows a cross-section of the intermediate cane obtained after the first stage of the drawing process. The structure remained almost identical to the original one, with just minor imperfections in some of the holes related to the cane preparation required for image acquisition. In the second stage of the drawing process, the cane was sleeved into a PMMA tube and drawn again to obtain the final mPOF. The resultant mPOF is shown in Figure 9b, where the SEM image of the microstructure was observed to have some degree of geometrical deformation. The observed distortion in the roundness and size of the air holes may have been due to the complex fibre preparation process necessary for the SEM image acquisition, in which hot razor blades were employed.

**Figure 10:**
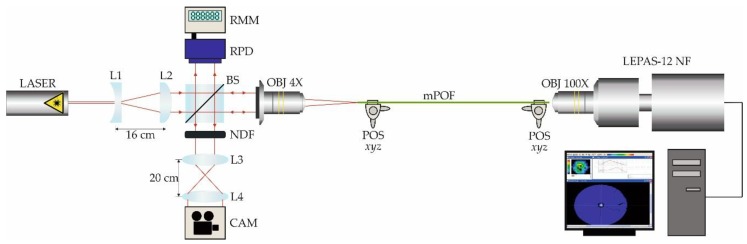
Schematic diagram of the experimental set-up used to measure the near-field pattern of a short mPOF sample. L1: plano-concave lens (*f*′ = −40 mm); L2: plano-convex lens (*f*′ = +200 mm); L3: symmetrical convex lens (*f*′ = +150 mm); L4: symmetrical convex lens (*f*′ = +50 mm); BS: beam splitter; NDF: absorptive neutral density filter; CAM: digital camera; OBJ: objective; POS *xyz*: *xyz*-micropositioner; RPD: reference photodetector; RMM: reference multimeter.

In order to characterise the spatial power distribution at the exit of the fabricated mPOF, the near-field pattern of a short sample (30 cm in length) was measured using a Hamamatsu LEPAS-12 Near-Field optical measurement system. The complete experimental set-up is shown in Figure 10, where a He–Ne laser source was precisely focused onto the fibre core in order to excite the guided mode and measure the corresponding near-field pattern of the light exiting the fibre. The 100× objective lens located at the entrance of the LEPAS system provided a resolution of 0.25 µm/pixel in the measured digital near-field image, allowing a detailed map of the light power distribution in the core/cladding region of the mPOF.

**Figure 11:**
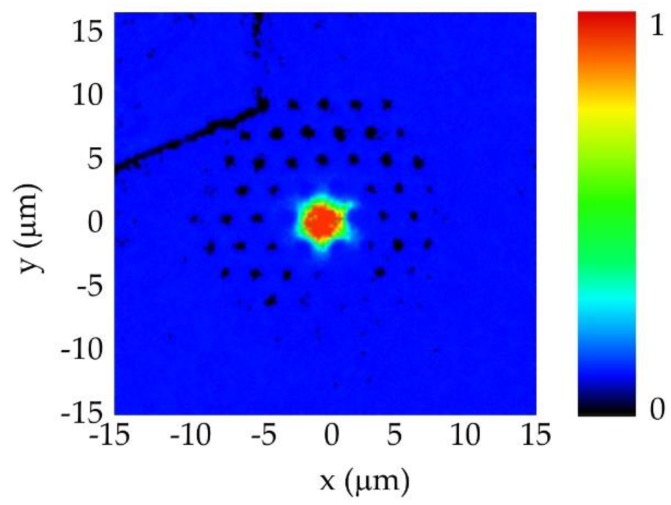
Near-field pattern at the mPOF output in a squared 30 × 30 µm^2^ region around the fibre core.

The experimental results of the near-field measurements are shown in Figure 11. It was clearly observed that most of the light was confined within the fibre core, following the same distribution pattern as that observed in the computer simulations (see Figure 8b).

## 4. Conclusions

In this work, we demonstrated that the use of through-coolant deep-hole drilling bits for the creation of PMMA holey preforms intended for the fabrication of microstructured polymer optical fibres is an excellent option in terms of fabrication time reduction and preform quality. It was proven that the time required for the preform drilling process can be greatly reduced if suitable drill bits and drilling parameters are chosen. Specifically, in the four-ring structure considered, the time required was reduced by one order of magnitude. In general, this result will also be fulfilled for any number of rings, since the average time to drill a hole is a constant independent of the number of rings considered. The quality of the holes drilled using different types of deep-hole drill bits was also analysed. The parameters chosen to evaluate the quality of the holes were the diameter error, cylindricity deviation, coaxiality deviation of the holes and the surface roughness of the inner walls. From their measurements, we concluded that through-coolant drill bits (SLD2, SLD3 and TCCD3) provide qualitatively better results than non-coolant drill bits (HSTD3). Regarding through-coolant drill bits, it is noteworthy that two design parameters must be taken into account: the diameter and the path geometry of the inner cooling holes, and the trajectory of the chip exit channel. Both parameters have a significant impact on the quality of the drilled holes. On the one hand, drill bits with straight cooling and chip exit paths (SLD) provided better quality parameter values than drill bits with helical paths (TCCD), where the cooling and chips flow in a helical trajectory. On the other hand, higher diameters in the cooling paths, and therefore higher refrigeration, provided better results in the measured quality parameters of the drilled holes. Finally, with the aim of validating the experimental work, we measured the near-field pattern of an mPOF fabricated employing the SLD3 drill bits, with the subsequent time reduction and quality improvement, and the results were excellent, in agreement with the theoretical simulations performed.

## Figures and Tables

**Table 1:** Selected drilling parameters for the different drilling bits.

Drill	Diameter (mm)	Length-To-Diameter Ratio	Coolant	Feed Speed (mm/min)	Rotation Speed (rpm)	Bit Length (mm)
HSTD3	3	40	External	-	1500	120
TCCD3	3	30	Internal (40 bars)	800	8000	93
SLD2	2	50	Internal (40 bars)	150	1500	110
SLD3	3	50	Internal (40 bars)	150	1500	175

**Table 2:** Comparison of the time required to create the structured preform using the different types of drill bit.

Drill	Time (min)	Preform Length (mm)
HSTD3	375	120
TCCD3	25	93
SLD2	70	110
SLD3	100	175
